# Retraction Note: Translation and psychometric evaluation of the Persian version of Knee Outcome Survey-Activities of Daily Living Scale (KOS-ADLS)

**DOI:** 10.1186/s12891-025-09200-y

**Published:** 2025-09-24

**Authors:** Hooman Minoonejad, Mohammad Amin Henteh, Roshanak Keshavarz, Mehdi Safarzadeh, Ali Montazeri

**Affiliations:** 1 Department of Sports Injury and Biomechanics, Faculty of Sport Sciences and Health, University of Tehran, Tehran, Iran; 2 Department of Physiotherapy, School of Rehabilitation, Tehran University of Medical Sciences, Tehran, Iran; 3 Population Health Research Group, Health Metrics Research Center, Iranian Institute for Health Sciences Research, Tehran, Iran; 4 Faculty of Humanity Sciences, University of Science and Culture, Tehran, Iran

**Retraction Note: BMC Musculoskelet Disord 24, 687 (2023)**

**https://doi.org/10.1186/s12891-023-06823-x**

The Editors have retracted this article. Following the publication of this article, concerns have been raised regarding permission for the Persian version of the Knee Outcome Survey-Activities of Daily Living Scale (KOS-ADLS) used in this study and ethics approval. Further investigation by the Publisher noted that the ethics approval mentioned in the study (IR.UT.SPORT.REC.1398.063) was not granted for this study, and this study was done without ethics approval. Additionally, the authors were unable to provide any evidence showing that permission for the use and translation of KOS-ADLS was given by the original developer.

Mohammad Amin Henteh and Ali Montazeri disagree with this retraction. None of the remaining authors has responded to the Publisher regarding this retraction.

